# Mechanisms of clay mineral-induced targeted deposition and synergistic CO_2_ sequestration potential in the CCUS-EOR process

**DOI:** 10.1371/journal.pone.0331674

**Published:** 2025-09-05

**Authors:** Miaoxin Zhang, Jingchun Wu, Liyuan Cai, Bo Li, Yang Zhao, Yangyang Hou, Fang Shi, Chunlong Zhang

**Affiliations:** 1 Key Laboratory for EOR Technology (Ministry of Education), Northeast Petroleum University, Daqing, China; 2 Daqing Yongzhu Petroleum Technology Development Co Ltd., Daqing, China; Dawood University of Engineering and Technology, PAKISTAN

## Abstract

**Background:**

Strongly water-sensitive reservoirs with high clay content face challenges in conventional development due to clay swelling and impeded seepage. CO_2_ injection shows potential for enhanced oil recovery (EOR) and carbon sequestration; however, the role of clay minerals in regulating CO_2_-induced asphaltene deposition and sequestration remains unclear.

**Methodology:**

We conducted experiments on clay-oil interactions, nuclear magnetic resonance (NMR), measurements of crude oil properties, and long core water flooding tests to evaluate deposition, reservoir damage, and CO_2_ sequestration.

**Conclusions/Significance:**

Results demonstrate that clay minerals significantly promote CO_2_-induced asphaltene deposition, with the deposition amount in clay-containing crude oil increasing by 37% compared to clay-free systems. The interfacial tension (IFT) between crude oil and CO_2_ decreases from 15.68 to 12.53 mN/m at 10 MPa with increasing clay content, while crude oil viscosity reduces by up to 43.58% when CO_2_ injection exceeds 30 mol%. Microscale NMR analysis confirms that clay-asphaltene aggregates preferentially block large pores, reducing reservoir heterogeneity and enhancing CO_2_ sequestration in medium/small pores. Macroscale long-core experiments highlight the significance of high clay mineral content for geological sequestration, showing that the CO_2_ sequestration rate increases from 43.15% to 48.21% as clay content rises from 8.35% to 29.92%. Although deposition slightly impairs permeability, it drives CO_2_ into medium/small pores, thereby achieving a balance between oil recovery efficiency and long-term storage stability.

## 1. Introduction

Strongly water-sensitive reservoirs characterized by high clay mineral content and high sensitivity to external fluids are inadequately developed through conventional water injection, which hampers the effective replenishment of formation energy [1]. Due to its physical properties, carbon dioxide is highly injectable, and it can solve the problem of hydration and expansion of clay minerals in the reservoir that block seepage channels [2].

Clay minerals can be categorized into two main types: crystalline and amorphous, based on their crystal shape. In oil and gas basins, crystalline lamellar silicates are the predominant clay minerals. Crystalline layered silicates are predominant in oil and gas-bearing basins. Common clay minerals include montmorillonite, illite, chlorite, mixed-layer clays (montmorillonite/chlorite, montmorillonite/illite), and sea chlorite. Intrusion and retention of foreign fluids, release and entrapment of clay particles, deformation of reservoir rocks, and expansion of clay minerals during the development of water-sensitive reservoirs can lead to reservoir damage. [3]. Reservoir injury results from the combined effects of particulate transport, fluid physicochemical reactions, and the deformation of field rocks [4–8].

Many studies have shown that CO_2_ injected into the reservoir will have complex physicochemical reactions with the rock and fluid. Firstly, it is possible that the CO_2_ injected into the reservoir can cause a complex physicochemical reaction with the fluid [9].CO_2_ injection has many advantages in the development of conventional sandstone reservoirs, mainly reflected in the reduction of crude oil viscosity, crude oil volume expansion, extraction, supplement formation energy, dissolution, and inhibition of clay expansion, and so on [10,11]. (1) Reduce the viscosity of crude oil: CO_2_ can be dissolved in the form of molecules in a large number of crude oil, so that the intermolecular forces between the crude oil weakened, the molecular activity of the molecules enhanced, to reduce the viscosity of crude oil [12], improve the flow capacity of crude oil. (2) Crude oil volume expansion: carbon dioxide dissolved in crude oil, with the increase in the amount of CO_2_ dissolved in crude oil and layer temperature, volume expansion of crude oil after the increase in kinetic energy [13], while the resistance to flow is reduced.(3) Extraction: When the pressure increases to a certain value after the light components of crude oil can be extracted by carbon dioxide, the oil phase will occur with the gas phase carbon dioxide mass transfer phenomenon, the light components and carbon dioxide to form a carbon dioxide-rich gas phase to improve the recovery rate [14].(4) Supplementary formation energy: With the continuous exploitation of oil reservoirs, there will be a phenomenon of rapid decline in formation pressure. CO₂ enables rapid replenishment of formation energy to maintain high formation pressure so that the crude oil has a high driving force. (5) Corrosion: CO_2_ dissolved in the formation of carbonic acid can be dissolved in the formation of water and rock in the carbonate and other minerals in a chemical reaction, causing the rock pores and throats to dissolve, expanding the pores and throats [15]. (6) Inhibiting clay expansion: low permeability reservoirs usually contain clay minerals, water injection and development of clay minerals are easy to absorb water and expansion [16], resulting in permeability decline, and CO_2_ can inhibit the expansion and dispersion of clay minerals, reducing the pore blockage of the reservoir.

In addition, CO_2_ oil stimulation can achieve the dual effect of improving crude oil recovery and carbon sequestration [17], which perfectly coordinates the relationship between resource recycling and environmental protection and achieves the important goal of sustainable development.

However, researchers pay less attention to the strong water-sensitive reservoir, and the role of clay minerals in solid-phase deposition mechanisms, reservoir damage, and oil recovery is underexplored. Therefore, it is urgent to quantitatively characterize the impact of solid deposition types on reservoir physical properties and determine the impact of clay minerals on asphaltene deposition and the main controlling factors of reservoir damage.

Currently, researchers have conducted a large number of studies on the development of gas injection in different types of reservoirs, explored the asphaltene generation law and the damage caused by the deposition to the reservoir, and explained the reasons for asphaltene deposition caused by CO_2_ drive and its impact on the development effect [18,19].

However, considering the diversity of CO_2_ sources, the CO_2_ content in the injected gas varies, so it is important to investigate the type of injected gas, the CO_2_ content, the injection pressure, and the injection rate on the asphaltene deposition law and its effect on the reservoir [18–20]

Asphaltene deposition in porous media will cause a change of wettability so that the hydrophilic reservoir will be changed to lipophilic, and the influence of rock mineral composition and fluid properties on wettability should be considered; the change of wettability may lead to poor development results. Experimental methods and models for asphaltene deposition have been continuously optimized based on long-term research and development [21]. Due to differences in reservoir physical properties, the deposition pattern of asphaltenes varies in different types of reservoirs. For example, modeling shale using the pore structure of nanofiltration membranes requires the evaluation of injection pressure, temperature, CO_2_ soaking time, and CO_2_ pore structure changes as determining factors of the instability of asphaltene deposition in nanoscale pores [22]. The asphaltene content in crude oil has a large impact on the asphaltene deposition pattern, and a small amount of CO_2_ injected into crude oil with a high content of asphaltene will disrupt the equilibrium state between reservoir fluids, resulting in the stripping of the colloid encapsulated in the outer layer of asphaltene. Therefore, controlling the injected CO_2_ content can reduce the amount of asphaltene deposition to a certain extent and obtain higher crude oil recovery [23,24]. Considering the variability of crude oil components, it is necessary to evaluate the amount of asphaltene deposition, components, and physical properties, and to explore the phase transition law between oil and gas under the consideration of temperature and pressure [25,26]. The presence of clay increases the polarity of the system, which leads to the adsorption of asphaltenes on the clay surface, resulting in an increase in the amount of asphaltenes deposited and a decrease in the porosity of the porous medium [27]. The interaction between brine and CO₂ induces salt precipitation, affecting the core’s physical properties. Higher salt precipitation after the interaction of high-salinity aqueous solutions with CO_2_ leads to a significant decrease in reservoir permeability due to deposition in the rock pores, whereas the effect is smaller at low salinity [28].

Solid-phase deposition (asphaltene, clay particles, and inorganic salts) is easily generated by the interaction with reservoir fluids during CO_2_ flooding, which can cause structural changes in the reservoir [29,30]. Therefore, it is important to consider the pattern of solid-phase deposition and the degree of core porosity change in the presence of clay minerals to improve the recovery rate further.

Existing studies on clay-asphaltene interactions in CO₂-EOR have primarily focused on conventional reservoirs with low clay content or non-water-sensitive formations, where clay minerals are often treated as passive components (e.g., [18,25]). However, in strongly water-sensitive reservoirs, clay minerals (especially montmorillonite) exhibit dynamic behaviors (swelling, migration, and surface adsorption) that may actively regulate CO₂-induced asphaltene deposition and pore structure evolution—an interplay rarely quantified. We hypothesize that: (1) Clay minerals promote CO₂-induced asphaltene deposition via electrostatic attraction (between negatively charged clay surfaces and polar asphaltene groups) and hydrogen bonding; (2) This targeted deposition (preferential blocking of large pores) reduces reservoir heterogeneity, thereby enhancing CO₂ sequestration in medium/small pores.

This study simulated the state of clay minerals when they are present in the fluid, investigated the effects of CO_2_ injection volume, injection pressure, and clay mineral content on the asphaltene generation pattern, and evaluated and analyzed the crude oil physical properties (interfacial tension, viscosity, and crude oil family composition). Based on the nuclear magnetic resonance (NMR) technique, the variations of core porosity and permeability were elucidated. Based on the correlation between organic solvent properties and sediment types, the effects of asphaltenes, clays, particulate matter, and inorganic salts on core properties were quantified, and the main sediment types that have a greater impact on porosity were identified, revealing the effects of targeted deposition on pore structure and the ability to modulate the non-homogeneity of reservoir pores. The results reveal the potential of CO_2_ injection in strong water-sensitive reservoirs for enhanced recovery and carbon burial, complement the mechanism of CO_2_ drive development, and have a certain reference value for the development of gas injection in other sensitive reservoirs.

Novelty: This study differs from previous work in three key aspects: (1) It focuses on highly water-sensitive reservoirs, a relatively underexplored system; (2) It quantifies the synergistic effects of clay minerals on asphaltene deposition and CO₂ sequestration, rather than treating these as independent processes; (3) It integrates data from the microscale (NMR pore characterization) and macroscale (long core flooding) to reveal the field-scale implications of the coupled mechanisms governing clay-asphaltene interactions.

## 2. Materials and methods

### 2.1. Materials

The experimental oil was based on the Methods of Physical Analysis of Fluids in Oil and Gas Reservoirs (GB/T 26981−2020) [26,31]. The formation of crude oil was formulated using on-site crude oil and simulated hydrocarbon gases, and the saturation pressure of the formation crude oil was 5.16 MPa, the ratio of dissolved gas to oil was 22.47 m^3^/m^3^, and the density of the formation crude oil under reservoir conditions (10.5 MPa, 40°C) was 0.803 g/cm^3^. The viscosity was 17.64 mPa·s, and the molecular weight of the formation crude oil was 505.14 g/mol. According to the analysis of the crude oil family composition, the content of saturated hydrocarbons, aromatic hydrocarbons, colloids, and asphaltenes in the crude oil was 43.5%, 28.5%, 11.2%, and 9.4%, respectively. The water used in the experiment was simulated formation water formulated from an on-site formation water recipe; the gas used in the experiment was CO_2_ with 99.9% purity and simulated dissolved gas; the average reservoir permeability in the study block was 13.84 × 10^−3^ μm^2^, and the water sensitivity index ranged from 88 to 97%, and the results of the basic physical property test showed that the permeability and porosity of the selected cores were similar. The bentonite clay used in this study has an average particle size of 1.0 µm, comprising 90% montmorillonite along with minor amounts of feldspar, calcite, quartz, and other minerals.

### 2.2. Experimental equipment

The main equipment includes high-temperature and high-pressure PVT and ISCO double-cylinder pumps (flow rate 0.001 ~ 107mL/min, maximum pressure 7500 psi), back-pressure and pressure-limiting pumps (maximum pressure 70.0MPa), and high-pressure non-magnetic core grippers (temperature upper limit of 80°C, pressure upper limit of 40MPa). Low-field magnetic resonator (MesoMR12-060H-I), magnetic field strength 0.3 T, resonance frequency 12 MHz. Liquid chromatograph (Prominence LC-20A), plunger capacity 10 μL, flow rate precision 0.06% RSD below. Gas-liquid separator (volume 20mL, accuracy 0.05mL), gas flow meter (accuracy 0.001mL), and high-pressure piston vessels.

### 2.3. Analysis of the physical properties of crude oil

Interfacial Tension Experiment [32]: (1) Load an oil sample into a high-pressure microsyringe and slowly squeeze a drop of oil into a cuvette filled with high-pressure gas so the drop is suspended at the end of the needle. (2) Adjust the focal length and angle of the instrument to capture the oil drop. (3) The shape parameters of the oil drop were extracted to calculate the oil-gas interfacial tension. (4) The experiment is repeated thrice, and the data is statistically analyzed.

Formation Crude Oil Viscosity Test [33]: (1) Transfer the crude oil in PVT to a falling ball viscometer and maintain the oil sample as a single-phase fluid. (2) Release the ball at three angles of 22.5°, 45°, and 67.5° and record the fall time. Repeat three times. (3) Calculate the viscosity from the formula and statistically analyze the data.

Determination of crude oil group components [34]: (1) According to the purpose of analysis and the characteristics of the sample, choose n-hexane-dichloromethane mixed solvent as the mobile phase solvent, etc. (2) The sample is injected into the high-performance liquid chromatography (HPLC) using a sampler. (3) The mobile phase carries the sample into the chromatographic column, and the components of each crude oil group are separated in the column, and the separated components are detected by the detector in turn. (4) The signals generated by the detector are transmitted to the processing, and the content of each crude oil group component is calculated by the corresponding quantitative method.

### 2.4. NMR experiments and blockage rate calculations

**Fig 1:**
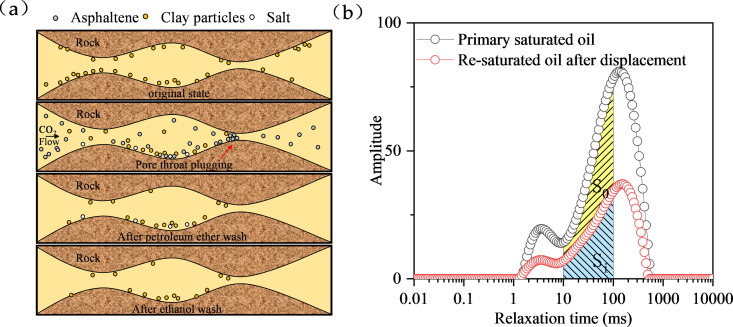
(a) Deposit washing process (b) Schematic of pore plugging calculation.

Nuclear magnetic resonance (NMR) is a technique for detecting pore structures by measuring the amplitude and rate of the NMR relaxation signal of hydrogen nuclei in pore fluids and mathematically inverting the feedback signal to create a T2 relaxation time spectrum [35–37]. The experimental procedure is shown in Fig 1 (a). The NMR experiments were performed using a MesoMR12-060H-I low-field magnetic resonator (Niumag Corporation, China) with a magnetic field strength of 0.3 T and resonance frequency of 12 MHz. Sample preparation: Core samples were saturated with formation brine (salinity: 10,000 mg/L) without additional solvents; no solvent signal suppression was used.Reference standard: Calibrated using water (δ = 4.79 ppm) at 25°C.Temperature: All experiments were conducted at 40°C (reservoir temperature).

(1) The core is scanned by NMR to calculate porosity after evacuation and saturation with brine. (2) The core was saturated with formation crude oil at a constant rate (0.01mL/min), scanned, and the initial oil saturation calculated.
(3) Inject carbon dioxide into the core at a constant flow rate of 0.1mL/min, and control the pressure of the return valve at 10MPa. Record the dynamic data changes during the replacement process, and stop the replacement when the gas-oil ratio is greater than 1500m3/t.
(4) Clean the core with an organic solvent, measure the porosity and permeability of the core again, and conduct NMR scanning of the core again under the condition of saturated formation oil.
(5) The process of calculating the degree of blockage of the pore throat for determining the pore size is shown in Fig 1 (b).

$$\text{b}=\frac{{\text{S}}_{\text{o}}}{{\text{S}}_{\text{o}}+{\text{S}}_{\text{i}}}\times \text{100}\%$$

Where b is the pore throat plugging rate, %; S0 is the difference between the initial saturated oil volume and the secondary saturated oil volume; Si secondary saturated oil volume.

### 2.5. Evaluation of the combined damage caused by different types of deposits

**Fig 2:**
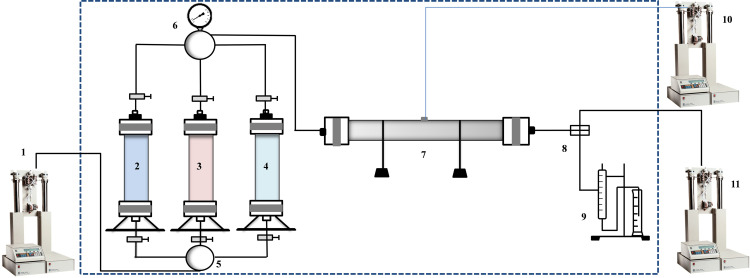
Experimental device process of the CO_2_ flooding experiment.

(1) Long cores spliced with natural cores from the target block were used for the high-pressure exfoliation experiments; refer to 2.4 for the specific experimental procedure.
(2) According to the characteristics of petroleum ether, toluene, and ethanol, the core was cleaned after expulsion, and the pore penetration coupler was used to measure the core in stages [38], as shown in Fig 2.
(3) Calculate the degree of change in pore penetration and clarify the effects of asphaltenes, clay minerals, and salt.

## 3. Results and discussion

### 3.1. Asphaltene deposition pattern of CO_2_ injection in crude oil

#### 3.1.1. Effect of CO_2_ injection volume.

**Fig 3:**
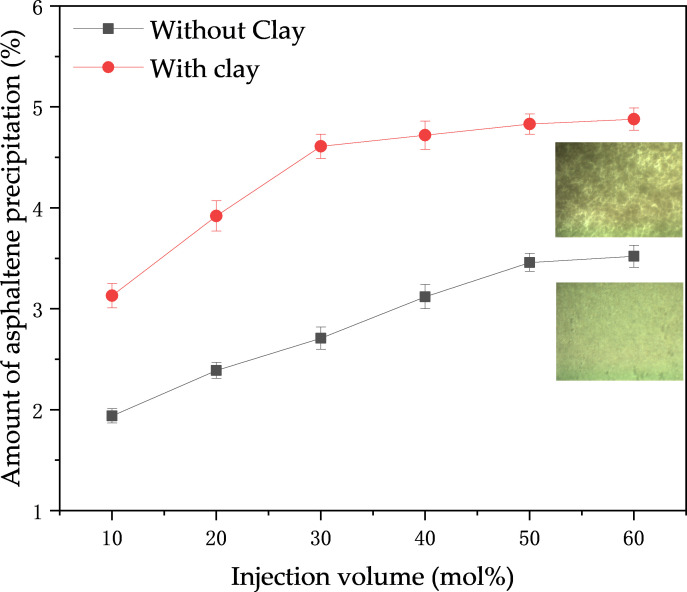
Effect of CO_2_ injection on asphaltene deposition.

Two realizations can be drawn by comparing the relationship of asphaltene precipitation under six different CO_2_ injection amounts, as shown in Fig 3. The first is that the amount of asphaltene precipitation in crude oil increases with the increase of the CO_2_ injection amount. When CO_2_ was not injected initially, asphaltene was uniformly and stably dispersed in the crude oil as micelles. The increase in asphaltene deposition slowed at a critical CO₂ injection level of 30 mol% due to the establishment of a new equilibrium between CO₂ and crude oil after asphaltene deposition under current pressure. Secondly, the asphaltene deposition in the clay-added crude oil increased significantly due to the negative charge on the surface of the clay minerals and the electrostatic interaction with the asphaltene, as well as the hydrogen bonding between the hydroxyl groups and the polar groups of the asphaltene. This trend exhibits similarity to previous research findings, with a consistent increasing trend, thereby validating the reliability of the mechanism by which clay promotes deposition [39].

#### 3.1.2. Effect of CO_2_ injection pressure.

**Fig 4:**
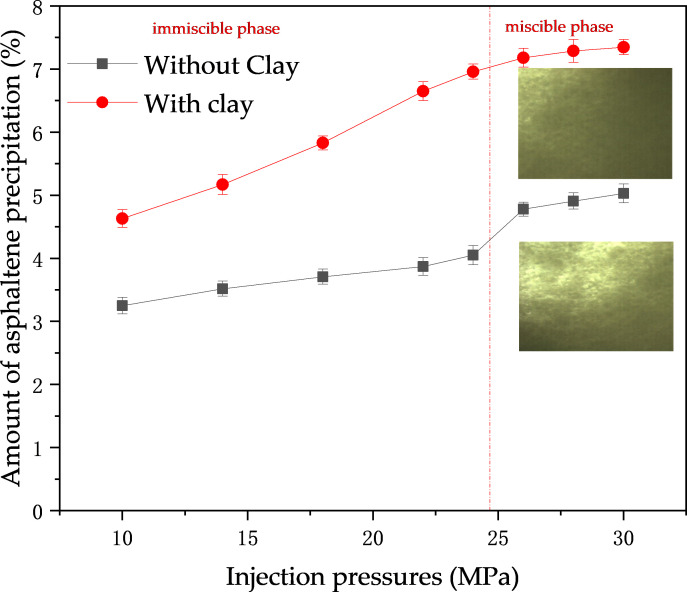
Effect of pressure on asphaltene deposition.

There were two main changes in asphaltene solid-phase deposition at different injection pressures: overall, the mass of solid-phase deposition increased with increasing injection pressure. The growth of asphaltene deposits increased significantly when the experimental pressure increased from the unmixed phase to the mixed phase, whereas it slowed down when the experimental pressure was greater than the mixed phase pressure. Secondly, comparing the effect of clay minerals, it was found that the trend of increase was the same, but the pressure was lower when there was a slowing trend in the amount of clay-containing crude oil deposited. This is shown in Fig 4.

#### 3.1.3. Effect of clay content.

**Fig 5:**
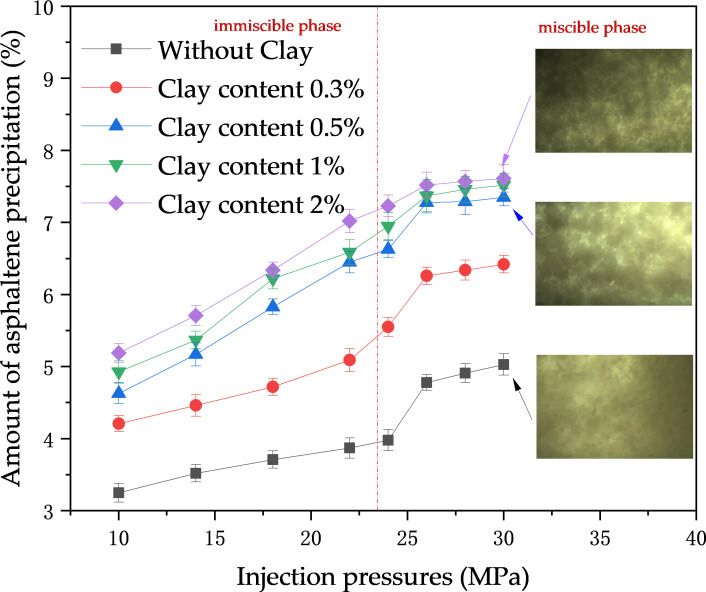
Percentage of sediment mass at different injection pressures.

The effect of different clay mineral contents on the trend of asphaltene deposition was explored. The experimental results are shown in Fig 5. Firstly, taking 10MPa as an example, it is observed that the amount of asphaltene deposition increases with clay mineral content, but the increase is small. Then the amount of increase in deposition was found to be much higher with miscible pressure (26 MPa) than with immiscible pressure (10 MPa), which was attributed to the fact that asphaltene deposition in crude oil by CO_2_ increased with increasing pressure, leading to an increase in adsorption on the surface of the clay minerals.

### 3.2. Changes in the physical properties of reservoir oils

#### 3.2.1. Change of interfacial tension.

**Fig 6:**
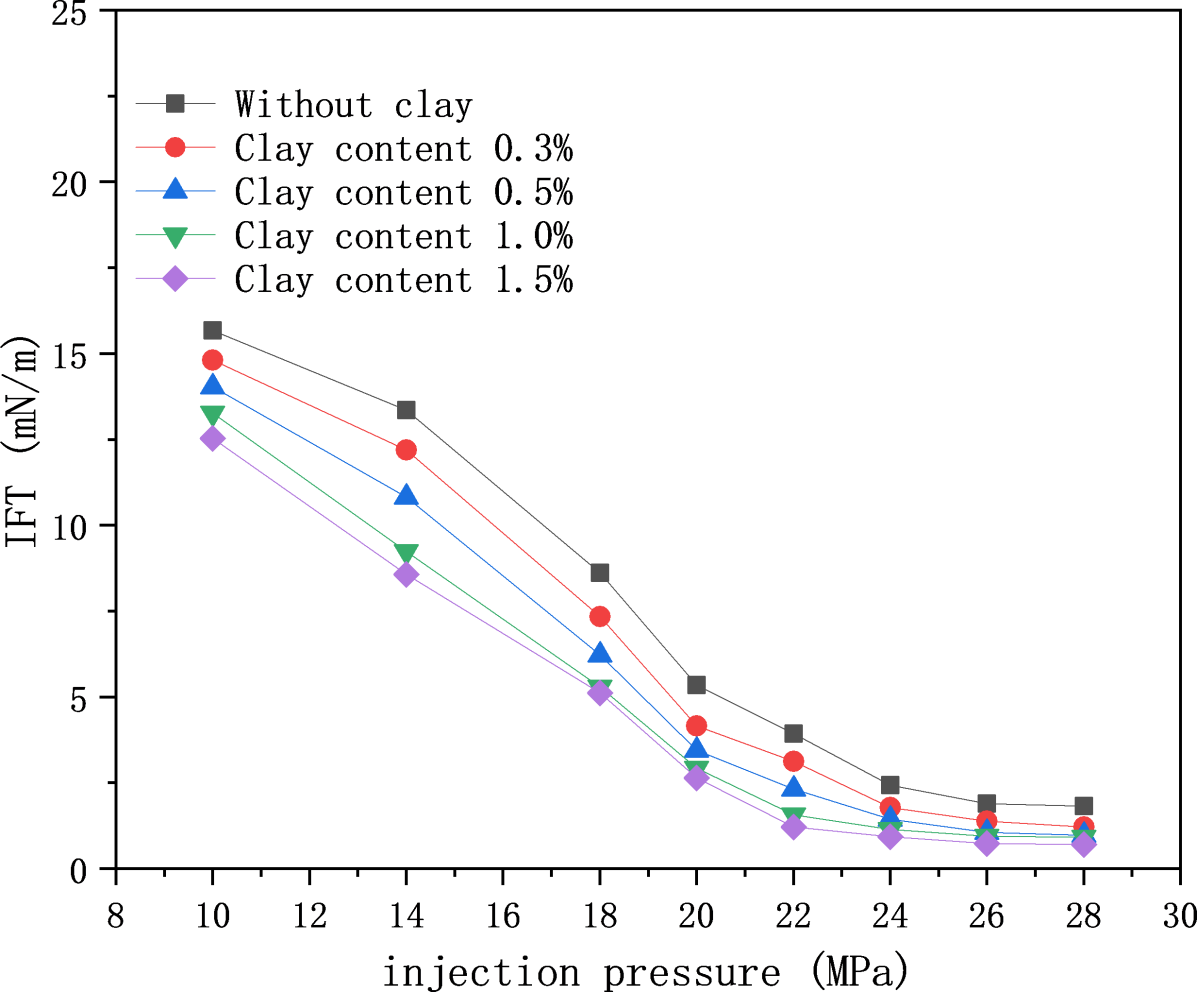
Effect of clay content on IFT.

The changing law of interfacial tension (IFT) between crude oil and CO_2_ was investigated by adding different amounts of clay minerals to crude oil, as shown in Fig 6. The clay mineral content during CO_2_ injection caused a difference in the interfacial tension between oil and gas. For example, at 10 MPa, the IFT of oil samples without clay and oil with 1.5% clay content were 15.68 and 12.53 mN/m, respectively, and the difference in oil-gas IFT diminished after reaching the miscible pressure [40]. The dynamic variation trend of IFT is consistent with the findings of previous studies, validating the field relevance of the laboratory results [41].

#### 3.2.2. Changes in crude oil viscosity.

**Fig 7:**
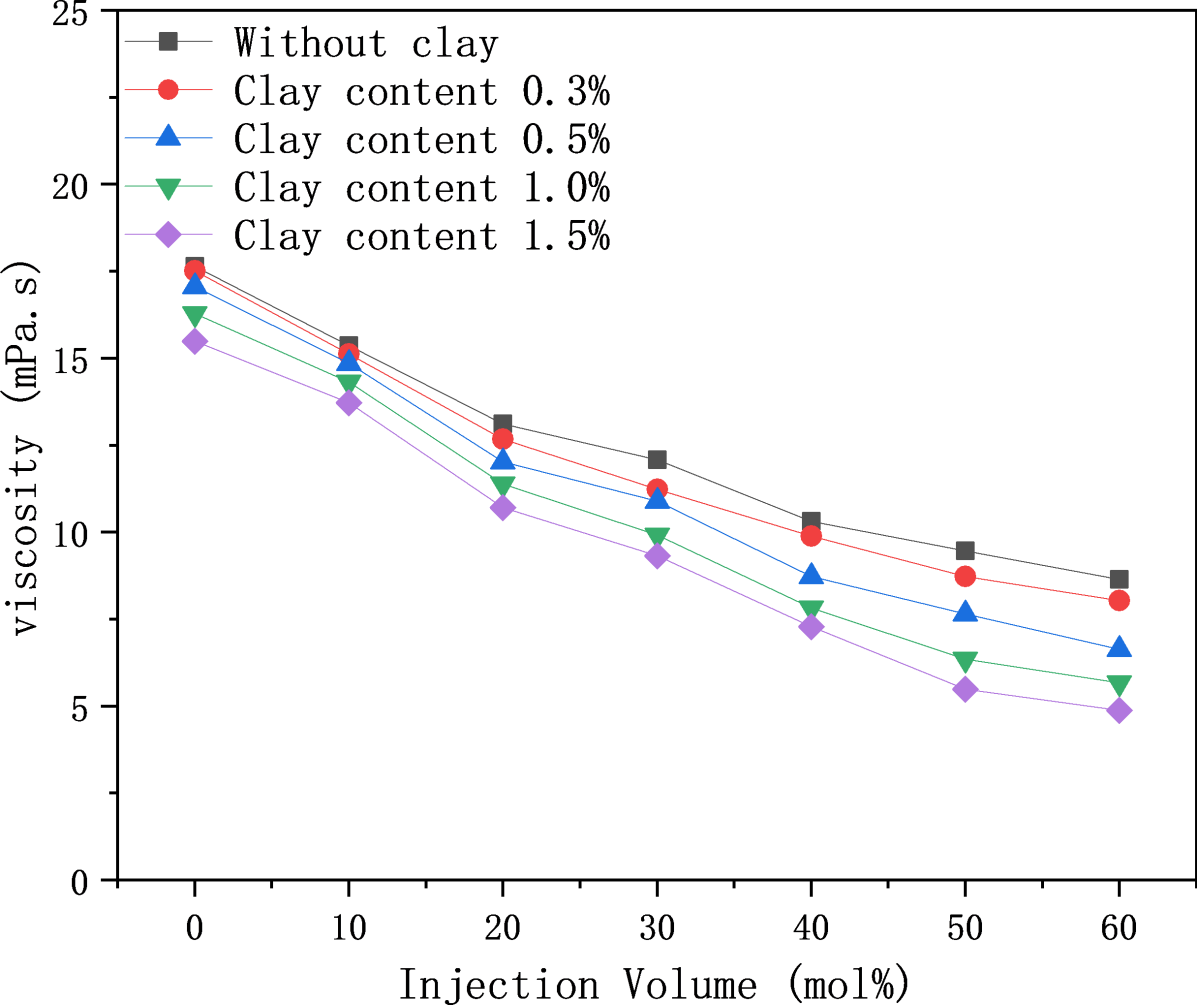
Effect of clay content on crude oil viscosity.

The viscosity of various oil samples with increasing CO_2_ injection was investigated using a high-temperature and high-pressure PVT and a falling ball viscometer. Two results can be summarised in Fig 7. The first is that the clay content in the absence of CO_2_ injection has a small effect on the crude oil viscosity (15.49–17.64 mPa.s). The viscosity difference between different oil samples increased after CO_2_ injection by more than 30 mol%. When the CO_2_ injection amount reaches 60 mol%, the viscosity difference ratio between clay-free and 1.5% clay-containing oil samples can reach 43.58%. Secondly, under the same pressure condition, the higher the clay mineral content, the lower the viscosity of the oil samples. This is due to the adsorption of clay minerals, leading to an increase in asphaltene deposition and a decrease in the proportion of heavy components in the oil phase.

#### 3.2.3. Changes in Crude Oil Group Components.

**Fig 8:**
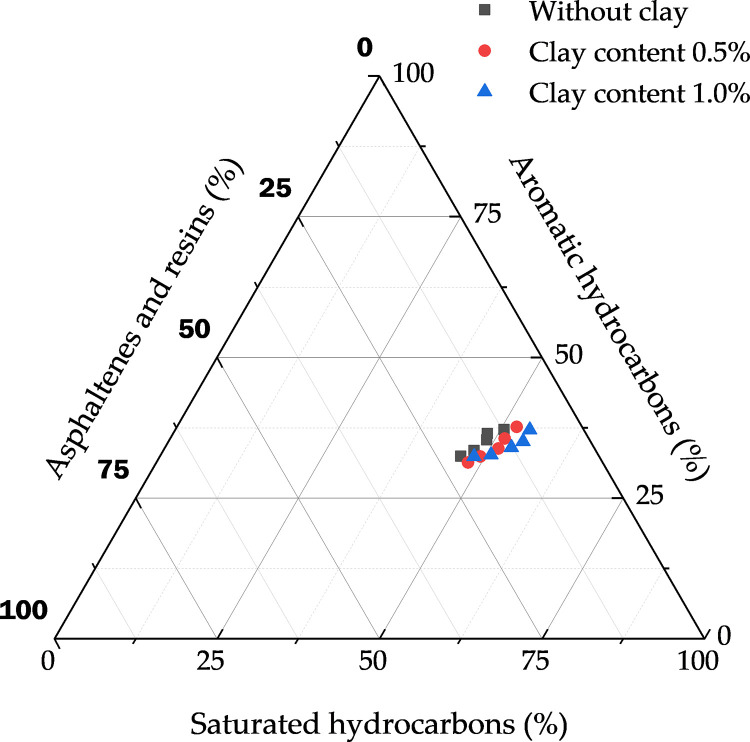
Effect of clay content on crude oil group components.

Considering the interaction between CO_2_ and different oil samples, the change rule of crude oil group components was detected by liquid chromatography, and three-phase diagrams were plotted after data normalization, as shown in Fig 8. It was found that the content of saturated and aromatic hydrocarbons both increased with the increase of CO_2_ injection, and the content of non-hydrocarbons (asphaltenes and colloids) decreased. The saturated hydrocarbon content of the oil samples with added clay minerals increased by about 4% after CO_2_ injection, the aromatic hydrocarbon changed less, and the non-hydrocarbon content decreased by about 3.8%. Asphaltenes in the crude oil are characterized by polycyclic aromatic cores with polar functional groups (e.g., hydroxyl, carboxyl, and pyrrolic groups), as confirmed by the observed changes in crude oil family composition. These polar groups contribute to strong intermolecular interactions, including hydrogen bonding and π-π stacking, which stabilize asphaltene micelles in the oil phase under initial conditions. However, upon CO₂ injection, the dissolution of CO₂ disrupts this stability, causing asphaltene aggregation—an effect amplified by clay minerals (e.g., montmorillonite) via electrostatic attraction between the clay’s negatively charged surface and the asphaltene’s polar groups [42].

### 3.3. Study on Deposition Characteristics Induced by CO₂ Injection and Carbon Sequestration Potential

#### 3.3.1. Effect of injection Pressure on reservoir Properties.

**Fig 9:**
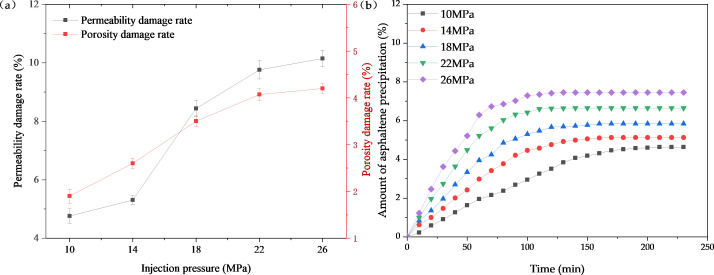
Evaluation of depositional damage (a) Variations in core physical properties (b) Temporal variation law of deposition amount.

To evaluate the effect of solid-phase deposits on core porosity and permeability, two indicators, porosity change rate and permeability damage rate (change rate of core porosity and permeability before and after CO_2_ drive), were used for the analysis [43]. Fig 9 (a) shows that the magnitude of the effect of solid-phase deposits on the reservoir increases with the increase in the injection pressure. The permeability damage rate increases from 5.31% to 10.15% as the displacement pressure rises from 14 MPa to 26 MPa. Fig 9(b) illustrates the relationship between asphaltene deposition amount and time under different pressures. It is observed that as the injection pressure increases, the deposition rate accelerates, and the time required for the deposition amount to reach stability is shortened.

**Fig 10:**
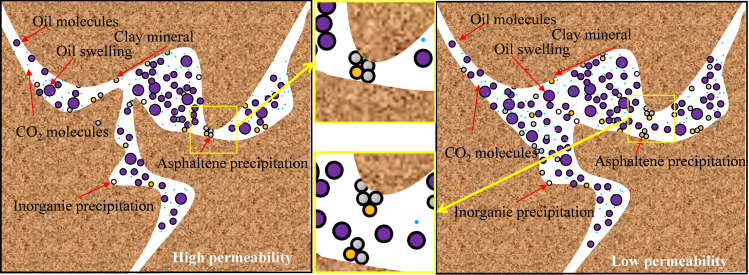
Schematic diagram of deposition plugging in pores in different reservoirs.

Solid-phase deposition causes more severe damage to permeability. Although the damage to porosity also shows an increasing trend, the overall variation is relatively small, mainly because the pore volume of the core is much smaller than its apparent volume. As shown in Fig 10, when the pore space is large, the interaction between CO₂ and crude oil is more sufficient, thus generating more deposition. In contrast, when the pore space is small, the amount of sediments is relatively small, but they tend to cause more severe pore plugging.

#### 3.3.2. Microscale Characterization of Pore Change Rate and CO₂ Sequestration Rate.

Based on the NMR scanning technology, we compare the pore structure changes in the core at different stages to clarify the solid phase deposition distribution and the degree of crude oil recovery. By analyzing the NMR T_2_ spectra under mixed-phase and non-mixed-phase injection pressures, the effects of clogging rate and the degree of crude oil mobilization in pore radii of different sizes were clarified.

**Fig 11:**
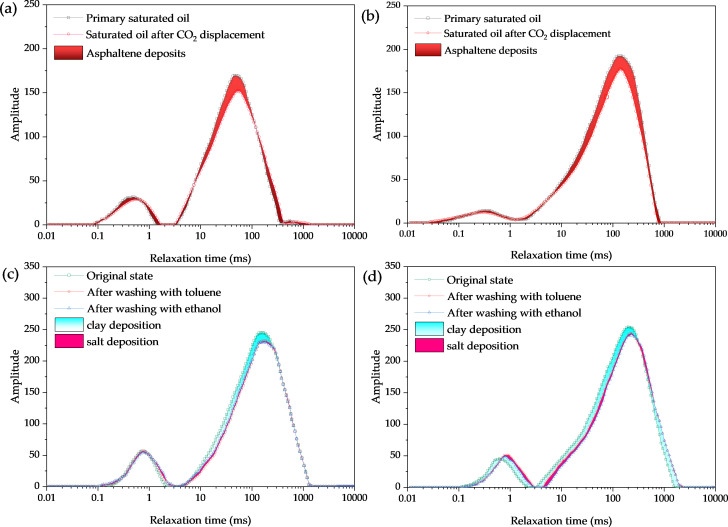
(a) and (b) NMR T_2_ spectra of asphaltene deposition under non-mixed-phase versus mixed-phase drive, respectively. (c) and (d) are the NMR T_2_ spectra of inorganic deposition under non-mixed-phase and mixed-phase driving, respectively.

According to the experimental results in Table 1, it is evident that asphaltene deposition and clay particle accumulation are the main controlling factors for pore blockage [44,45]. Three insights can be drawn. Firstly, overall, as the pore size decreases, the influence of clay minerals increases, the influence of asphaltene relatively weakens, and the effect of salt precipitation on pore throat blockage is relatively small. As the injection pressure of CO_2_ and crude oil increases, reaching a mixed phase state, CO_2_ has a stronger ability to extract light components, asphalt precipitation increases, and the pore blockage rate sharply increases. The second aspect is the proportion of sediment in the pores. In immiscible driving, the influence of asphaltene on the pores is higher than that of clay mineral deposition. This is because the degree of stripping clay minerals by immiscible carbonate is lower than that of miscible driving, so the damage caused by asphaltene deposition is higher. Under miscible phase driving, the influence of asphaltene and clay minerals is similar. This is because the clay minerals in the pores are stripped to a greater extent and form larger aggregates with asphaltene through adsorption, depositing in large pores. The third aspect is that the salt content in the aqueous solution is relatively low, and the interaction time between CO_2_ and the core is relatively short; the impact of salt precipitation on the pores is minimal. As shown in Fig 11 (Schematic diagram of precipitation plugging in pores in different reservoirs), solid-phase deposits exhibit distinct plugging patterns in reservoirs with varying physical properties, which further explains the observed changes in porosity and permeability.NMR results confirm the correlation between pore-throat structure and deposits [46]. This finding is consistent with previous studies, and our research further quantifies the impact of other types of deposits.

**Table 1:** Recovery, sequestration, and plugging efficiency of pores with different sizes.

Injection pressure	Hole throat size	recovery efficiency (%)	sequestration rate (%)	Pore blockage rate (%)	Percentage of influencing factors (%)		
Asphaltene	Clay	Salt
14MPa	Large	32.06	42.15	4.46	61.88	35.65	2.47
	Medium	24.75	32.16	2.38	57.14	40.76	2.10
	Small	15.56	8.38	0.59	55.93	42.37	1.69
23MPa	Large	43.41	39.46	10.63	50.33	46.66	3.01
	Medium	36.59	35.13	7.75	49.42	47.10	3.48
	Small	32.08	16.74	2.34	46.15	51.28	2.56

The results show that the pore blockage rate affects the microscale storage rate of carbon dioxide. With the increase of pressure, the blockage of large pores decreases, while that of medium and small pores increases. This is because the main blocking space of solid deposits is large pores, which weakens the heterogeneity of the reservoir and further increases the amount of CO₂ entering medium and small pores. The CO₂ sequestration rates of large, medium, and small pores in the immiscible state are 42.15%, 32.16%, and 8.38% respectively, with an overall sequestration rate of 42.15%. In the miscible state, the sequestration rates in large and medium pores slightly increase, while that in small pores significantly increases to 16.74%.

### 3.4. CO₂-induced Targeted Deposition Mechanism and CO₂ Sequestration Performance

Based on high-pressure displacement experiments on long rock cores, we conducted experimental research on targeted deposition of rock cores with different clay contents. We compared the changes in porosity and permeability of rock cores at different locations before and after CO_2_ injection, clarified the degree of damage caused by solid-phase deposits to the reservoir under flowing conditions [47–50], quantified the degree of damage to rock properties caused by sediment types such as asphaltene, inorganic salts, and clay minerals. This study revealed the relationship between clay minerals and deposits, as well as the CO₂ sequestration potential of cores with different clay contents.

**Fig 12:**
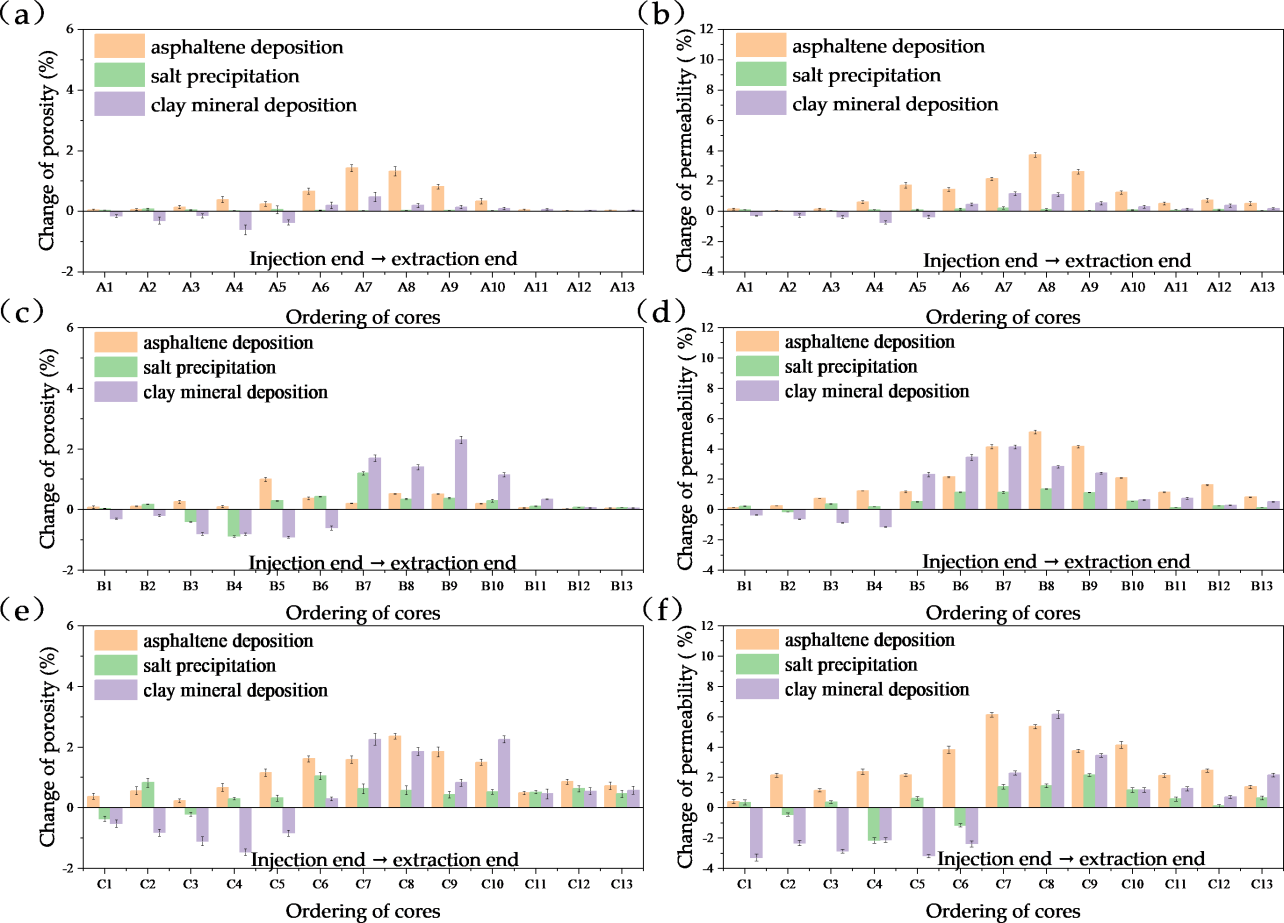
Changes in porosity and permeability of cores (with average clay contents as follows: (a)-(b) 8.35%, (c)-(d) 17.26%, and (e)-(f) 29.92%).

The experimental results are shown in Fig 12, and three conclusions can be drawn. Overall, due to the damage to core porosity, although it also shows an upward trend, the overall change value is relatively small, mainly because the pore volume of the core is much smaller than the apparent volume of the core, so the degree of porosity change is relatively small. The results show that with the increase of clay mineral content, the damage of solid deposition to core permeability is greater, and the main deposition zone expands. This is because the clay particles in the core pores are eroded by carbonic acid, leading to the spalling, migration, and adsorption of asphaltene, which is easier to form large aggregates, accumulate, and block in the pores, resulting in the reduction of the pore radius of the seepage channel.

From the perspective of clay mineral content, increased clay content exacerbates sediment-induced damage to core physical properties due to clay minerals’ larger specific surface area and stronger adsorption capacity, forming larger asphaltene deposits. Clay minerals hinder in-situ asphaltene deposition initially, causing migration toward the extraction end until aggregates grow too large to pass through pores. Spatially, higher clay content delays and expands deposition damage areas. Low-clay cores show damage concentrated in A6–A9, while medium/high-clay samples exhibit main deposition in B6–B10 and C4–C12, with increased clogging degree and range. Carbonic acid strips clay particles from pore throats, especially in the first half of the core. Low reservoir brine salinity results in minimal inorganic salt precipitation and pore throat damage.

Practical implications of CO₂ sequestration in high-clay reservoirs: The observed increase in sequestration rate (from 43.15% to 48.21% with clay content rising to 29.92%) highlights their potential as strategic storage sites. Field-scale application could leverage this by prioritizing such reservoirs for CCUS-EOR, as higher clay content enables more efficient CO₂ trapping per unit volume—critical for meeting carbon emission reduction targets.

Comparisons of CO₂ sequestration rates in cores with varying clay mineral contents have demonstrated that reservoirs with high clay mineral content are potential CO₂ sequestration sites, enabling the synergistic effect of enhanced oil recovery and efficient CO₂ storage, as shown in Fig 12. The results indicate that the overall CO₂ sequestration rate increases with the rise in clay mineral content, attributed to the larger specific surface area of clay minerals, which enhances their CO₂ absorption capacity. The core with low clay mineral content (8.35%) exhibited a CO₂ sequestration rate of 43.15%, while the core with high clay mineral content (29.92%) achieved 48.21%, representing an overall increase of 5.06% in CO₂ sequestration rate. The core displacement experimental results are consistent with the range of previous data, validating the field relevance of the laboratory results [51].

Storage integrity is enhanced by two key mechanisms: (1) Preferential blocking of large pores by clay-asphaltene aggregates (confirmed by NMR T2 spectra in Fig 10) reduces the risk of CO₂ channeling through major seepage channels; (2) The high specific surface area of montmorillonite (accounting for 90% of clay minerals) strengthens the physical adsorption of CO₂, stabilizing long-term storage. These effects collectively mitigate leakage concerns, a key constraint for industrial-scale sequestration.

**Fig 13:**
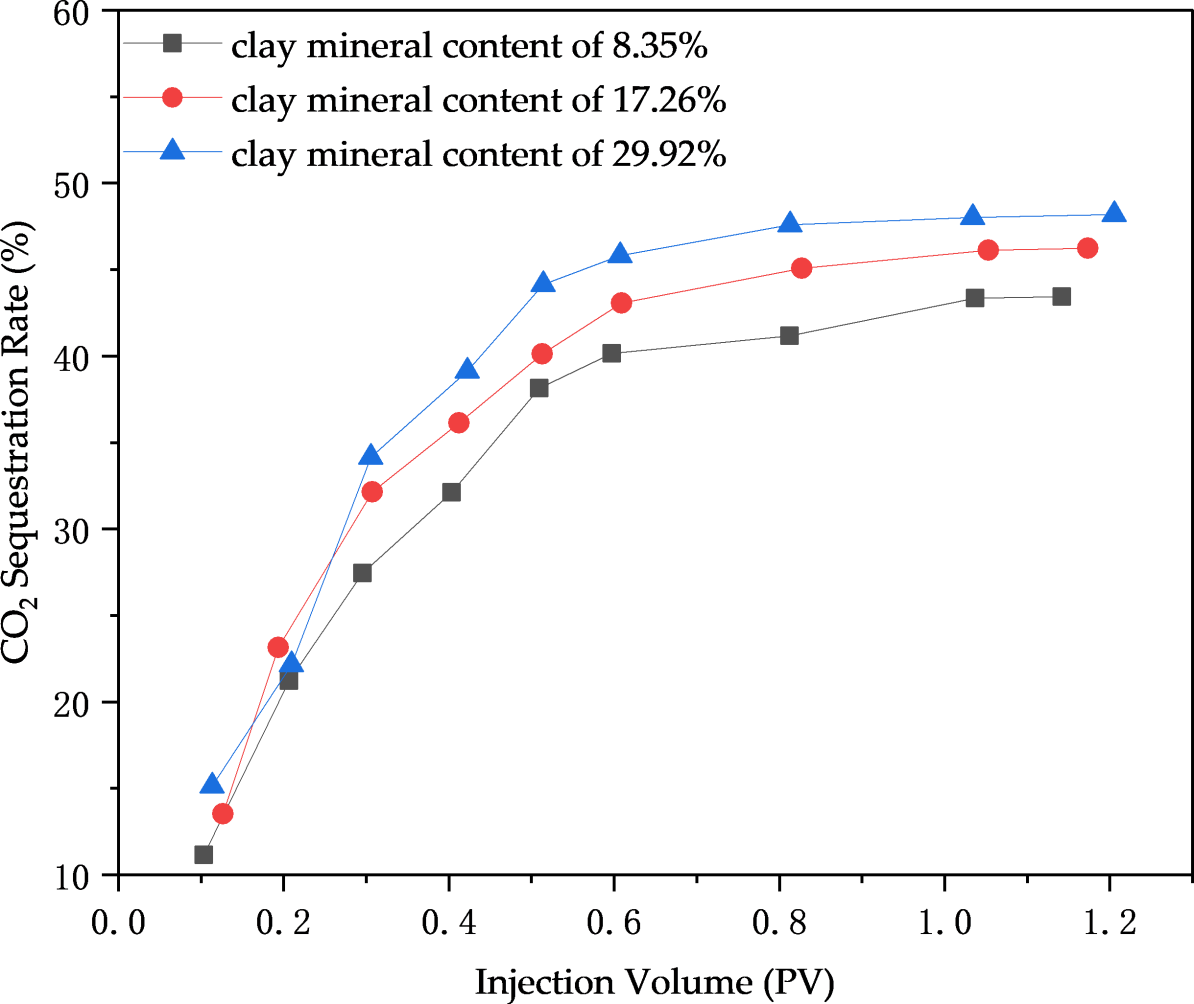
CO_2_ sequestration rates in cores of different clay minerals.

Notably, the synergistic relationship between asphaltene deposition and sequestration bridges EOR and carbon storage: While deposition slightly impairs permeability (Fig. 12), it redirects CO₂ to medium/small pores where trapping is more durable, balancing oil recovery efficiency with long-term storage stability, as show in Fig 13.

## 4. Conclusions

The CO_2_ displacement mechanism is studied using a CO_2_ displacement experiment in the strongly water-sensitive reservoir. The influence of clay minerals on asphaltene deposition was evaluated. Natural cores were used to explain the sedimentary distribution characteristics from the micro and macro scales, quantify the plugging degree of different types of deposits in the pores, and determine the changes in core physical properties in different regions. The specific conclusions are as follows:

(1) Experiments have confirmed that clay minerals promote asphaltene deposition, with the deposition amount increasing with CO₂ injection volume, pressure, and clay content. The asphaltene deposition in clay-containing crude oil is 37% higher than that in clay-free systems.
(2) Fluid property experiments show that increasing clay content reduces the interfacial tension (IFT) of crude oil, decreasing from 15.68 mN/m to 12.53 mN/m at 10 MPa. When the CO₂ injection volume exceeds 30 mol%, the viscosity of clay-containing crude oil decreases by up to 43.58%, the saturated hydrocarbon content increases by 4%, and non-hydrocarbons decrease by 3.8%.
(3) Nuclear magnetic resonance (NMR) scanning technology reveals that deposits preferentially block large pores, while CO₂ is mainly sequestered in large and medium pores. At 14 MPa, the plugging rates of large, medium, and small pores are 4.46%, 2.38%, and 0.59%, respectively, and the carbon sequestration rates are 42.15%, 32.16%, and 8.38%, respectively.
(4) Long-core displacement experiments with different clay contents indicate that higher clay content intensifies the impact of solid-phase deposition on core physical properties. The CO₂ sequestration rate increases with clay content, reaching 48.21% when the clay content is 29.92%, confirming that asphaltenes and clay minerals are the main controlling factors.

This study has limitations: laboratory conditions do not fully replicate in-situ reservoir complexity (e.g., long-term geochemical reactions); findings focus on montmorillonite-dominated clays, with limited generalization to other types (e.g., illite); and short-term experiments (<72 hours) do not capture long-term (>1 year) aggregate evolution.

Future work will address these via: long-term tests (≥6 months) to monitor pore plugging and sequestration stability; expanding to diverse clay types to generalize mechanisms; and coupling experiments with numerical simulations for field-scale validation.
